# Psychometric testing of the Determinants of Salt-Restriction Behaviour Questionnaire in people of Chinese ancestry: a methodological study

**DOI:** 10.1186/s12912-022-01124-5

**Published:** 2022-12-02

**Authors:** Alex Chan, Leigh Kinsman, Sally Wai-chi Chan

**Affiliations:** 1 School of Nursing and Midwifery, University of Newcastle, Newcastle, Australia; 2 School of Nursing, University of Wollongong, 33 Moore Street, Liverpool, NSW 2170 Australia; 3 La Trobe Rural Health School, La Trobe University, Melbourne, Australia; 4 Tung Wah College, Hong Kong SAR, China

**Keywords:** Diet, Salt, Salt reduction, Chinese, Diasporas, Questionnaire, Psychometric testing, Methodological study

## Abstract

**Purpose:**

Nurses play a key role in educating people about a salt-reduced diet to prevent or manage hypertension or cardiac failure. Assessment tools such as the Chinese Determinants of Salt-Restriction Behaviour Questionnaire (DSRBQ) can provide essential evidence to inform education strategies. This study aimed to translate the DSRBQ into English and evaluate the psychometric properties of the Chinese and English versions for people of Chinese ethnicity in Australia.

**Methods:**

A two-phase cross-sectional descriptive study was conducted. Phase 1: The questionnaire was translated into English using the back-translation method. The translation equivalence and content relevance were evaluated by an expert panel. Three items were revised and eight items were removed. Phase 2: Internal consistency and stability of the questionnaires were evaluated by a group of Chinese Australians.

**Results:**

Both the English and Chinese versions had satisfactory psychometric properties. In phase 2, 146 participants completed the questionnaire (test), and 49 participants completed the retest. The Cronbach’s alpha scores were 0.638 and 0.584 respectively, and the overall intra-class correlation coefficients were 0.820 and 0.688 respectively for the English and Chinese versions. The Item-Content Validity Index (CVI) ranged from 0.50 to 1.00. The Scale-CVI was 0.94.

**Conclusion:**

The DSRBQ has been translated into English. Both English and Chinese versions have acceptable validity and reliability. The tools can be used in people from a Chinese cultural background living in Australia. Further validation testing may allow the tools to be adapted for use with other Chinese diaspora groups. The validated DSRBQ will support the development of evidence-based salt reduction nursing assessment tool and interventions for Chinese diasporas who reside in a country where Chinese cultural practices are employed by a minority.

## Introduction

High dietary salt consumption is a global health issue and the cause of many non-communicable diseases including hypertension and renal disease [1, 2]. Dietary salt restriction is an effective primary and secondary preventive measure for hypertension [3–5]. Currently, the World Health Organization (WHO) recommends a maximum daily salt consumption for healthy adults of 5 g (g) per day [1]. However, many adults consume higher than the recommended amount in their daily diets [6, 7]. This may be associated with cultural practices and personal food taste preferences.

A sociology study in 2011 estimated that more than 40 million individuals of Chinee descent lived in 148 countries around the world [8]. The 2016 Census found that over 5.1% (1.2 million) of Australians reported Chinese ancestry, and of these, only 25% were born in Australia [9]. An Australian study showed that Chinese immigrants who had resided in Australia for a longer period had more cardiovascular risk factors such as hypertension, diabetes and high cholesterol [10]. For example, the prevalence of hypertension among Chinese female immigrants who had lived in Australia for 30 years or longer was greater compared with those who had lived in Australia for less than 10 years (prevalence ratio = 1.47; *p* < 0.05) [10].

Recent statistics showed that average salt consumption in China was above 10 g/day [11], in Singapore it was 8.5 g/day [12], in Italy it was 9.0 g/day [13] and in Australia it was 9.6 g/day [14]. It is important to note that most salt consumption studies have focused on the main ethnic groups of the countries in which the studies were conducted. The dietary salt practices and related public health issues in minority population groups such as migrants are less explored. Further, the effects of Westernisation on food selections may influence non-Westerners’ dietary selections, leading to an increase in overall salt consumption. In general, dietary practices are often affected by multiple factors including personal preferences and cultural inheritance. Therefore, it is important to study the salt-related knowledge and perceptions of and perceived barriers to following a low-salt diet of the target Chinese population group.

Evidence showed that there was a close relationship between salt consumption and blood pressure [15]. With an increase of 2.5 g (1 g of sodium) in daily salt consumption increased the blood pressure by 2.11/0.78 mmHg and this effect was more significant in people who consumed more than 7.5 g of salt per day [15]. A recent literature review in 2022 suggested that salt-related health education might have positive influences on dietary behavioural changes and Chinese diasporas especially the new migrants might experience linguistic and cultural barriers when they seek dietary advice in their host countries [16]. Little is known about the current landscape and effects of the existing salt-related education and the challenges of reducing salt consumption for preventing hypertension among Chinese diasporas. Given more than 1.2 million (5.1%) Australians reported Chinese ancestry and that salt reduction is an effective preventive strategy for hypertension, there is a pressing need to assess the level of daily salt consumption, perceptions and knowledge in this ethnic group, and whether they continue the dietary practices learnt in their homeland or partially Westernise their meal patterns. The purpose of this study was to translate the original Determinants of Salt-Restriction Behaviour Questionnaire (DSRBQ) from Chinese to English and adapt it to the Chinese Australian context. The original DSRBQ was used in a series of salt restriction behavioural studies in Beijing, China (*n* = 403 [17], *n* = 513 [18] and *n* = 799 [19]). Overall, it has good reliability (Cronbach alpha = 0.757) and validity (accumulative contribution rate = 63.5%). However, it has not yet been adapted and translated into other languages. So, a validated tool is available to investigate the Chinese Australians’ salt-related behaviour and barriers to modify their salt consumption. The findings will inform the healthcare providers in the design of further salt reduction strategies for this population group.

## Methods

### Design

**Fig. 1:**
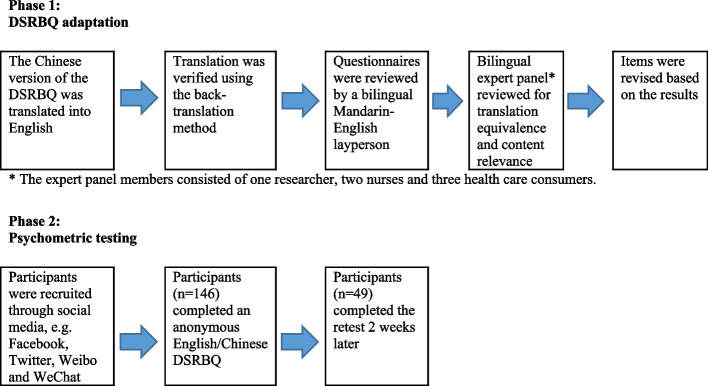
Flow diagram of the study design. Abbreviation: DSRBQ, Determinants of Salt-Restriction Behaviour Questionnaire

This psychometric methodological study was conducted in Australia. It had two phases: Phase 1, Determinants of Salt-Restriction Behaviour Questionnaire (DSRBQ) adaptation; and Phase 2, Psychometric testing of both the revised Chinese and translated English DSRBQs (see Fig. 1). The cross-cultural adaptation process was guided by previous psychometric testing studies conducted in Asian Pacific Region [20–22] and the STrengthening the Reporting of OBservational studies in Epidemiology (STROBE) checklist for cross-sectional studies was used to guide the preparation of this report [23].

### Instrument

The original Chinese DSRBQ consists of 3 parts. Parts 1 and 2 include 34 items (Table 1):

1. Demographic characteristics (*n* = 9), including age, gender, ethnicity, education, marital status, employment, income and health conditions.
2. Personal dietary practice (*n* = 12):
  3 items measured using categorical variable scales
  5 items measured using 5-point Likert scales ranging from ‘daily (1) to never (5)’, ‘never (1) to always (5)’ and ‘very light (1) to very salty (5)’
  3 items requiring participants to directly answer the number of meals being consumed at home and the percentage of food consumed at home vs away from home.
  1 binary scale (yes/no) item.
3. Salt-related health education/medical advice that the participant has received (*n* = 7), measured using a binary scale (yes/no).
4. Salt-related health knowledge (*n* = 6) measured using categorical variable scales.

Part 3 includes 47 items forming six subscales:

1. Perceived threat (*n* = 5)
2. Knowledge/perceived susceptibility to and severity of the disease (*n* = 6)
3. Perceived benefits of action subscale (*n* = 3)
4. Perceived benefits of using a measuring spoon (*n* = 3)
5. Likelihood of following the recommended interventions (*n* = 10)
6. Perceived barriers (*n* = 20).

All items are measured using a 5-point Likert scale ranging from strongly disagree (1) to strongly agree (5).

### Phase 1: Adaptation of the Determinants of Salt-Restriction Behaviour Questionnaire

The Brislin’s model for translation and validation of instruments was used to guide the translation of the DSRBQ [24]. The original Chinese questionnaire [19] was translated from Chinese to English by an experienced Chinese-English interpreter, and accuracy was confirmed using the back-translation method by an author (AC). Discrepancies, mostly in terminologies such as the word selections for sauce, condiment and paste, were amended by the author. The revised versions were reviewed by an independent bilingual Mandarin-English layperson to ensure the questionnaires were accurately translated and written with an appropriate level of literacy.

#### Expert panel review for translation equivalence and content relevance

The translation equivalence and content relevance were evaluated by a panel of experts between September and October 2019. Australia is home to many migrants from different countries and administrative divisions. Chinese people in Australia come from China, Hong Kong, Taiwan, Vietnam, Malaysia and Singapore. To promote the accuracy of the validation, the expert panel members were Chinese-English bilingual and purposively invited from different regions in Asia. They consisted of one researcher, two nurses and three health care consumers. They were recruited through the authors’ professional networks. An independent research assistant sent a personalised invitation letter to the potential experts to invite them to participate in the study.

#### Translation equivalence evaluation and content validity

The panel members were invited to evaluate the translation equivalence and content relevance of each item across the Chinese and English versions. Firstly, the translation equivalence of each item was evaluated by using a four-point scale ranging from 1 = not equivalent to 4 = most equivalent. Any items that received a score of 1 or 2 by more than 20% (*n* = 1) of the panel members were reviewed [21] and revised. Secondly, the expert panel members used a four-point scale (1 = not relevant, 2 = somewhat relevant, 3 = relevant, and 4 = most relevant) to evaluate the content relevance of each item to the Chinese Australian culture and community. The Content Validity Index (CVI) is the most common approach for content validity in questionnaire development and adaptation [22]. For each item in the questionnaire, an Item-level CVI (I-CVI) was calculated by dividing the number of panel members who scored 4 (very relevant) by the total number of panel members (*n* = 6) [22]. Then, a Scale-level CVI (S-CVI) would be calculated by using the mean I-CVI, which was the sum of all I-CVIs divided by the total number of items (*n* = 81) [20]. A S-CVI score of 0.80 (80%) or higher is considered as having a good content validity [20–22].

### Phase 2: Psychometric testing of the revised Chinese and translated English Determinants of Salt-Restriction Behaviour Questionnaires

The reliability and validity of both the Chinese and English versions of the DSRBQ were evaluated through a cross-sectional descriptive study. Participants were invited to complete an anonymous English or Chinese DSRBQ, either online or paper-based, twice at an interval of two weeks. Similar answers should be obtained in the two tests if the tool has a high test–retest reliability [25].

#### Participants and setting

Participants were recruited through social media such as Facebook, Twitter, WeChat and Weibo from January to March 2020 and from July to November 2020 using a convenience sampling method. Participants completed either the Chinese or English questionnaire according to their preferences. Data collection was suspended between March and July 2020 due to the coronavirus (COVID-19) pandemic in Australia, when people were overwhelmed by COVID-19-related health information and lockdown. The inclusion criteria were: a) adults over 18 years old of Chinese ancestry; and b) those who had lived in Australia for at least 6 months. Adults who were unable to read a Chinese or English questionnaire were excluded from the study.

In general, there are a variety of recommended sample sizes for test–retest reliability. The recommended sample size ranged from 50 to over 1000 subjects or the item to response ratio was from 1:3 to 1:20 [26]. Perneger et al. [27] noted that a sample size of 30 could achieve a power of 80% to detect a problem that occurs in 5% of population. In this study, it was assumed that the null hypothesis value was 0.00, and the study aimed to achieve a power of 80% in a two-tailed test. Therefore, the minimum sample size for each version needed to be 50 or greater to detect a Kappa of 0.40 [28]. The same sample size determination was used in Girard et al. [25]. It is important to acknowledge that a small sample size may under identify problems with a questionnaire [27]. Given the data collection was heavily affected by the first global wave of the COVID-19 pandemic, the target sample size of at least 50 participants for each version was the most practical and appropriate option for Phase 2 study at that time.

#### Data analyses

The Statistical Package for the Social Sciences version 25 was used for data analysis. The level of significance was set at 0.05 for all tests. To minimise bias in data analysis, any questionnaires with more than 10% of the items missing were excluded from the analysis [29].

For test–retest reliability, Pearson Correlation and McNemar tests were used to examine the association and consistency in response to the continuous and nominal variables/items in Parts 1 and 2 of the questionnaires in the first test (T1) at week 0 and retest (T2) at week 2. Intraclass correlation coefficient (ICC) was used to examine the six Likert subscales in part 3. Any subscales with a low reliability value (i.e. less than 0.5) are considered to have poor reliability [30].

The internal consistency reliability was measured by Cronbach’s alpha to identify the homogeneity of the items in the questionnaire (Parts 2 and 3). An alpha of 0.65–0.80 was considered satisfactory [31]. In addition, the item-to-total correlation test was used to assess the internal consistency of the knowledge assessment questions in part 2.

### Ethical considerations

This descriptive cross-sectional study was approved by the Human Research Ethnics Committee at the University of Newcastle, Australia in which the study was conducted (approval number: H-2019–0180), and permission to use the questionnaire was obtained from the author of the DSRBQ (Chen et al., 2014). In Phase 1, expert panel members were assured of their confidentiality and signed a written informed consent form. All identifiable personal information was removed. Informed consent to participate was assumed by participants in Phase 2. Prior to completing the anonymous questionnaire, participants could choose to receive a paper copy or electronic copy of the participant information statement, which detailed the purpose and aims of the study. Participants were asked to indicate their consent to the study by ticking a box at the beginning of the questionnaire. On the completion of the questionnaire, if participants chose to be included in a draw to win one of three $100 gift vouchers, they were asked to enter their contact details in a separate database so that their responses could not be identified.

## Results

### Phase 1 – Translation equivalence and content relevance

Any items rated not equivalent or somewhat equivalent (scores of 1 or 2) in the Chinese-English translation by at least 20% of panel members were reviewed. The I-CVI was used to measure the content relevance of the questionnaire to Chinese Australians in both the English and Chinese versions. The I-CVI ranged from 0.50 to 1.00. A total of six items were scored below 0.80, the acceptable CVI level. Before the questionnaire modifications, the S-CVI was 0.94 for both versions, demonstrating the validity content of the DSRBQ.

Based on the results, three items related to demographic information were modified as they were designed according to the living environment and style in China, such as weekly income in Chinese currency (Renminbi). Eight items were removed from the questionnaire as they specifically asked about the salt reduction program in Beijing, China, e.g., non-standard Chinese cooking and dietary practices across the Great China Region such as consuming the sauce left in the pan after pan-frying was irrelevant to the Chinese Australian lifestyle. After the items (*n* = 8) were removed from both versions, the S-CVI was 0.95.

#### The adaptation of DSRBQ

Seventy-three items remained in the questionnaires, which consisted of:

- nine demographic items; nine personal dietary practice items; four items about the salt-related health education/medical advice that the participant had received in part 1,
- six salt-related health knowledge items in part 2, and
- forty-five Likert scale items (part 3).

The internal consistency and reliability of the revised questionnaires were validated in Phase 2. Table 1 shows the differences between the original and revised DSRBQ versions.

**Table 1:** The differences between the original and revised DSRBQ versions

	**Original DSRBQ**	**Revised DSRBQ**	**Remarks**
Part 1	A total of 28 items: • Demographic characteristics (*n* = 9), including age, gender, ethnicity, education, marital status, employment, income and health conditions • Personal dietary practice (*n* = 12): • 3 items measured using categorical variable scales • 5 items measured using 5–point Likert scales ranging from ‘daily (1) to never (5)’, ‘never (1) to always (5)’ and ‘very light (1) to very salty (5)’ • 3 items requiring participants to directly answer the number of meals being consumed at home and the percentage of food consumed at home vs away from home • 1 binary scale (yes/no) items • Salt–related health education/medical advice that the participant has received (*n* = 7), measured using a binary scale (yes/no)	A total of 22 items: • Demographic characteristics (*n* = 9), including age, gender, place of birth, education, marital status, employment, income and health conditions • Personal dietary practice (*n* = 9): • 3 items measured using categorical variable scales • 3 items measured using 5–point Likert scales ranging from ‘daily (1) to never (5)’, ‘never (1) to always (5)’ and ‘very light (1) to very salty (5)’ • 2 items requiring participants to directly answer the number of meals being consumed at home and the percentage of food consumed at home vs away from home • 1 binary scale (yes/no) items • Salt–related health education/medical advice that the participant has received (*n* = 4), measured using a binary scale (yes/no)	• 6 items (Items 8, 13,14, 22, 26 and 27) were removed from the questionnaire because: • Item 8 was not relevant to the Chinese Australian lifestyle (I–CVI = 0.50) • Items 13 and 14 were related to the cooking style. However, the practices vary according to the participants’ background. Chinese people from different parts of Asia may interpret the questions differently • Items 22, 26 and 27 (I–CVI = 0.67). The questions were not related to the salt reduction strategies in Australia • Item 3 was changed from ‘ethnic groups in China, eg. Han, Zhuang, Hui, etc.’ to the ‘place of birth’ • Item 6 – the weekly income scale was changed and aligned with the 2011 Census, Personal Income Ranges (ABS, 2016) • Item 11 – the words ‘Beijing local government’ was removed from the answer options
Part 2	• Salt–related health knowledge (*n* = 6) measured using categorical variable scales	• Salt–related health knowledge (*n* = 6) measured using categorical variable scales	• No change
Part 3	A total of 47 items forming six subscales: • Perceived threat (*n* = 5) • Knowledge/perceived susceptibility to and severity of the disease (*n* = 6) • Perceived benefits of action subscale (*n* = 3) • Perceived benefits of using a measuring spoon (*n* = 3) • Likelihood of following the recommended interventions (*n* = 10) • Perceived barriers (*n* = 20)	A total of 45 items forming six subscales: • Perceived threat (*n* = 5) • Knowledge/perceived susceptibility to and severity of the disease (*n* = 6) • Perceived benefits of action subscale (*n* = 3) • Perceived benefits of using a measuring spoon (*n* = 3) • Likelihood of following the recommended interventions (*n* = 10) • Perceived barriers (*n* = 18)	• Two Likert scale items (Items 28 and 29) were removed because they were related to the measuring spoon given by the local government in Beijing, China

*Abbreviations*: *I-CVI* item-level CVI, *S-CVI* scale-level CVI

### Phase 2

#### Characteristics of the participants

The participants’ demographic data are shown in Table 2. A total of 152 participants attempted to complete the questionnaire in Week 0 (T1). Six participants’ responses were excluded from the analysis because they had missed more than 10% of the items. So, a total of 146 participants (English version: *n* = 67; Chinese version: *n *= 79) were included in the Week 0 analysis. A total of 49 participants (English version: *n* = 29; Chinese version: *n *= 20) took part in the retest (T2) in Week 2. Although a reminder was sent to all participants (*n* = 152) via email at the beginning of Week 2, some participants did not complete the questionnaire a second time.

**Table 2:** Demographic characteristics of the participants

Characteristics	Total sample (T1) (*n* = 146), n (%) [English: *n* = 67, Chinese: *n* = 79]	Retest (T2) (*n* = 49), n (%) [English: *n* = 29, Chinese: *n* = 20]
Gender		
Male	49 (33.6%)	14 (28.6%)
Female	97 (66.4%)	35 (71.4%)
Age: mean (SD)	35 (12.9)	39 (16.8)
Hypertension		
Diagnosed hypertension	14 (9.5%)	7 (14.3%)
Normotension	121 (82.3%)	39 (79.6%)
Unknown	12 (8.2%)	3 (6.1%)
Marital status		
Married	59 (40.4%)	16 (32.7%)
Single/separated/divorced	87 (59.6%)	33 (67.3%)
Place of birth		
Mainland China	45 (30.8%)	13 (26.6%)
Hong Kong	58 (39.7%)	20 (40.8%)
Taiwan	16 (11.0%)	8 (16.3%)
Other, e.g. Malaysia, Singapore	27 (18.5%)	8 (16.3%)
Education level		
Primary	2 (1.4%)	1 (2.0%)
Secondary	19 (13.0%)	6 (12.3%)
Tertiary or university	125 (85.6%)	42 (85.7%)
Occupation		
Unskilled	28 (19.2%)	11 (22.5%)
Skilled	31 (21.2%)	7 (14.3%)
Semi–professional/professional	15 (10.3%)	2 (4.1%)
Student	20 (13.7%)	6 (12.2%)
Self–employed	11 (7.5%)	5 (10.2%)
Unemployed/retired	41 (28.1%)	18 (36.7%)
Household income (per annum)		
< $20,800	11 (7.5%)	6 (12.2%)
$20,800–$41,599	17 (11.7%)	7 (14.3%)
$41,600–$64,999	32 (21.9%)	11 (22.4%)
$65,000–$103,999	31 (21.2%)	9 (18.4%)
> $104,000	52 (35.6%)	14 (28.6%)
No response	3 (2.1%)	2 (4.1%)

*Abbreviation*: *SD* standard deviation

Overall, the mean age of participants in the total sample in T1 was 35 years (SD = 12.9) and 39 years (SD = 16.8) in T2. Most participants were females (66.4% in T1 and 71.4% in T2) and 82.3% (*n* = 121) in T1 and 79.6% (*n* = 39) in T2 had no medical history of hypertension. About a third of participants (35.6%, *n* = 52) had annual household incomes over $104,000, which exceeds the median gross household income ($88,452) in 2017–2018 [32]. The demographic characteristics of the participants in both T1 and T2 groups were similar (Table 4). There were no significant differences in demographic data between the English and Chinese version groups.

#### Internal consistency and reliability

##### Parts 1 and 2 – Personal dietary practice, salt-related health education/medical advice and knowledge items

Analysis of all responses to the questionnaires showed satisfactory test–retest reliability in responses to most of the continuous and nominal items in Parts 1 and 2 over a 2-week period (Table 3). The test–retest reliability of both the Chinese and English versions was similar. Items 11 to 15 in Part 1 demonstrated poor to no associations with or correlations between T1 and T2 in both versions.

Table 4 presents the mean, SD, item-to-total correlations and overall Cronbach’s alpha for part 2 if a single item was deleted from the questionnaire. The Cronbach coefficient alpha of the total scores (showing internal consistency) was 0.638 for the English version and 0.584 for the Chinese version.

**Table 3:** The test–retest reliability of items in Parts 1 and 2

	**Question**	**Test**	**English version**	**Chinese version**
**Part 1**				
Q8a	Meals consumed at home: Weekdays–meals/day	**Pearson Correlation**	*r* = 0.111; *p* = 0.552	*r* = 0.367; *p* = 0.101
Q8b	Meals consumed at home: Weekends–meals/day	**Pearson Correlation**	*r* = 0.352; *p* = 0.061	*r* = 0.079; *p* = 0.740
Q9	Taking antihypertensive drugs	**McNemar**	1.000	0.625
Q10	Using a measuring spoon at home	**McNemar**	0.508	0.500
Q11a	Additional food seasoning–pickles	**McNemar**	0.035	0.008
Q11b	Additional food seasoning–sauces	**McNemar**	0.026	0.009
Q11c	Additional food seasoning–MSG/chicken essence	**McNemar**	0.000	0.001
Q11d	Additional food seasoning–soy sauce	**McNemar**	0.009	0.000
Q11e	Additional food seasoning–Nil	**McNemar**	0.002	0.000
Q12	Preference for a salty taste in food	**Pearson Correlation**	*r* = 0.275; *p* = 0.121	*r* = –0.174; *p* = 0.450
Q13	Amount of food consumed at home (%)	**Pearson Correlation**	*r* = 0.330; *p* = 0.070	*r* = 0.138; *p* = 0.550
Q14	How often uses a measuring spoon when cooking	**Pearson Correlation**	*r* = 0.004; *p* = 0.986	*r* = –0.221; *p* = 0.349
Q15	Deliberately limiting salt being added to cooking (Chinese version only)	**Pearson Correlation**	Not performed	*r* = –0.080; *p* = 0.739
Q16a	How do you add salt to cooking–use a spatula	**McNemar**	0.143	1.000
Q16b	How do you add salt to cooking–cooking pot	**McNemar**	0.035	0.754
Q16c	How do you add salt to cooking–tablespoon	**McNemar**	0.007	0.508
Q16d	How do you add salt to cooking–measuring spoon	**McNemar**	0.687	1.000
Q17a	Use of measuring spoon–work out the amount for each dish	**McNemar**	0.625	1.000
Q17b	Use of measuring spoon–follow the experience	**McNemar**	0.410	0.180
Q17c	Use of measuring spoon–follow the preferred taste	**McNemar**	0.001	1.000
Q17d	Use of measuring spoon–never use a spoon	**McNemar**	0.064	0.180
Q18	Has relatives with hypertension	**McNemar**	0.344	0.344
Q19	Advised by a doctor to reduce salt intake	**McNemar**	1.000	0.625
Q20	Advised by family, friend and relatives to reduce salt intake	**McNemar**	0.289	0.791
Q21	Advertising on TV, magazines and internet regarding salt reduction	**McNemar**	0.581	0.754
**Part 2**				
Q1	The amount of salt intake recommended by the World Health Organization	**McNemar**	0.267	0.500
Q2	The long–term impact of high salt intake on health	**McNemar**	1.000	0.581
Q3	The diagnostic criteria for hypertension	**McNemar**	1.000	0.388
Q4	The causes of hypertension	**McNemar**	0.625	1.000
Q5	The prevention of hypertension	**McNemar**	0.424	0.625
Q6	The complications of poor hypertension control	**McNemar**	0.581	0.687

*Abbreviations*: *r* correlation coefficient, *p* probability value

**Table 4:** Means, Standard Deviations, item-to-item correlation coefficients and overall Cronbach’s alpha for items in Part 2 if single items are deleted

**Part 2**	**Mean**		**Standard Deviation**		**Item–to–total correlation coefficient**		**Cronbach’s alpha if item is deleted**	
Item	English version	Chinese version	English version	Chinese version	English version	Chinese version	English version	Chinese version
Item 1 What is the maximum daily salt consumption for an adult recommended by the WHO?	1.261	1.100	0.443	0.302	0.207	–0.060	0.636	0.616
Item 2 A long–term high dietary salt intake can directly lead to…	1.630	1.425	0.486	0.497	0.283	0.241	0.611	0.533
Item 3 What are the diagnostic criteria for high blood pressure?	1.308	1.425	0.465	0.497	0.268	0.267	0.615	0.518
Item 4 What are the causes of high blood pressure?	1.862	1.888	0.348	0.318	0.503	0.488	0.527	0.428
Item 5 How can you prevent high blood pressure?	1.908	1.900	0.292	0.302	0.502	0.445	0.542	0.450
Item 6 What are the complications of poor blood pressure control?	1.785	1.725	0.414	0.449	0.485	0.451	0.522	0.410

*Note*: The overall Cronbach coefficient alpha of the total scores was 0.638 for the English version and 0.584 for the Chinese version

##### Part 3 – Likert items

The internal consistency and test–retest reliability for six subscales and the overall scale are presented in Table 5. Cronbach’s alpha coefficients for the overall scale showed that the English version had high internal consistency reliability of 0.816, with a value of 0.692 for the Chinese version in T1. Cronbach’s alpha for the six subscales ranged from 0.660 to 0.896 in the English version and from 0.657 to 0.923 in the Chinese version in T2, indicating adequate internal consistency. The ICC for the subscales ranged from 0.558 to 0.816 in the English version and from 0.421 to 0.818 in the Chinese version. The overall ICC results indicated that both the English (0.820) and Chinse versions (0.688) had good test–retest reliability over a 2-week period.

**Table 5:** Internal consistency and test–retest reliability for items in Part 3 for the overall scale and subscales at initial test (T1) and retest (T2)

**Part 3**	**Cronbach’s alpha**				**ICC** ^**a**^	
	English version		Chinese version		English version	Chinese version
	T1 (*n* = 67)	T2 (*n* = 29)	T1 (*n* = 79)	T2 (*n* = 20)		
Overall scale	0.816	0.786	0.692	0.829	0.820	0.688
1. Perceived threat subscale	0.874	0.876	0.928	0.756	0.560	0.421
2. Knowledge/perceived susceptibility/ severity to the disease subscale	0.828	0.866	0.829	0.899	0.816	0.681
3. Perceived benefits of action subscale	0.874	0.835	0.916	0.923	0.558	0.511
4. Perceived benefits of using a measuring spoon subscale	0.771	0.660	0.803	0.794	0.671	0.655
5. Likelihood of following the recommended interventions subscale	0.850	0.869	0.817	0.886	0.798	0.818
6. Perceived barriers subscale	0.783	0.896	0.765	0.657	0.762	0.652

*Abbreviations*: *ICC* intraclass correlation coefficient

^a^ICC – Intraclass correlation coefficient, *p* = 0.05 (two-tailed)

## Discussion

Findings from this study show that both the revised Chinese and translated English versions of the DSRBQ [19] are valid and reliable tools for use with individuals of Chinese ancestry in Australia. The rigorous translation and psychometric-properties-testing ensured the cultural equivalence of the questionnaires. In phase 1 – the Translation equivalence and content relevance study, a total of six items were scored below the acceptable I-CVI level (0.80). As a result, the items were either revised or removed from the questionnaire including the items related to the salt reduction program in Beijing, China. The S-CVI was 0.95 after eight items were removed from the questionnaire, which indicated the DSRBQ had excellent content validity overall [33].

In general, a scale with a Cronbach’s alpha in the range of 0.65 to 0.80 is considered as having an acceptable level of internal consistency [31], but some studies accept lower alpha values of 0.45 and above [34]. Therefore, in the present study, both versions have acceptable levels of internal consistency (0.638 and 0.584 for the English and Chinese versions respectively for the items in Part 2) and test–retest reliability (0.820 and 0.688 for the English and Chinese versions respectively for the items in Part 3). In consonance with the theoretical framework of HBM, all key subscales demonstrated strong correlations and test–retest reliability over time.

In comparison with the previous validation study [18], our study successfully recruited a unique group of participants, who were young (mean = 35, SD12.9) and single (59.6%, *n* = 87) and had no hypertension-related medical conditions (*n* = 121, 82.3%). Of this group, 18.5% (*n* = 27) of the participants were born outside the Greater China region, such as the United Kingdom or Malaysia. The average age of the participants in our study was more than 20 years younger than participants in the previous study in China. As Chen & Liao [18] recommended, preventive options could be offered to those who are relatively young but at risk of hypertension due to high dietary salt consumption. So, the participants in our study are highly likely to benefit from measures for the primary prevention of hypertension. This validation study showed that both the Chinese and English questionnaires have acceptable psychometric properties, indicating that the questionnaires are suitable and appropriate for this ethnic group, and the findings can inform nurses of the specific dietary-salt-related health behaviour risks and barriers for individuals of Chinese ancestry when they modify their dietary practices. 

The validated questionnaire has general demographic items followed by items about self-awareness of hypertension and health behaviour associated with dietary salt intake. The item-to-total correlation was used to measure the correlations between an item and the total score [35]. The acceptance range of the correlation value is from 0.3 to 0.7 [35]. The item-to-total correlation showed that items 1 to 3 in Part 2, which are related to knowledge of the daily maximum salt consumption, long-term health effects of salt and diagnostic criteria for hypertension, were below 0.3 in both versions. Although there was a marked improvement in the Cronbach’s alpha results when these three items were deleted, these items should be accepted and considered as very sensitive indicators for testing salt-related knowledge. This is because most participants could not correctly answer the essential knowledge items, and some of the responses were ‘I don’t know’. According to a previous psychometric testing study [36], people from different areas may have different levels of salt-related knowledge. Our participants were from very diverse backgrounds, which also reflected on the composition of the Chinese Australian population group. From a content point of view, if people do not know the recommended daily salt intake amount, hypertension diagnostic criteria and impact of salt on human health, they would not perceive excessive salt intake to be a health threat or take action to minimise the risks of hypertension. They are more likely to continue their current dietary salt habits, and adherence to maintaining a low-salt diet is likely to be suboptimal. Therefore, items 1 to 3 in Part 2 should remain in the questionnaire.

Cronbach coefficient alpha and ICC were used to assess the internal consistency and test–retest reliability for the Likert items (Part 3). All six subscales in both versions exceeded the recommended Cronbach alpha value of 0.70 at T1 [35, 37]. At T2, two subscales (perceived the benefits of using a measuring spoon in the English version and perceived barriers in the Chinese version) had Cronbach alpha values of 0.660 and 0.657 respectively. Although these values were below the recommended 0.70, the values were still above the acceptable level of 0.65 [31]. These results indicate strong overall internal consistency of the full scale. The ICCs for each subscale in Part 3 exceeded the suggested value of 0.40 [38]. Therefore, all Likert items/subscales were deemed to have sufficient test–retest reliability.

Construct validity was not conducted in this study. This was because no similar instruments could be used to compare with the findings from DSRBQ. The original DSRBQ has been rigorously validated and extensively used in China. It has good reliability (Cronbach alpha = 0.757) and validity (accumulative contribution rate = 63.5%). Gunawan & Marzilli [26] reported that construct validity may not be necessary if the translation process was accurate. In this study, the back-translation method and a translation equivalence evaluation completed by expert panel members were used to ensure the translation accuracy. So, the authors believed that the adapted DSRBQs had adequate construct validity.

In summary, the findings from this study indicate that both the Chinese and English versions of the DSRBQ [18] have sufficient validity and reliability to measure the dietary salt-related knowledge and health behaviours of people of Chinese descent. This questionnaire has the potential to identify the educational needs of clients as well as the roles of nurses in promoting and educating, using salt restriction as a method for primary and secondary prevention of hypertension for people of Chinese descent who are living in the Western world. Some health behavioural items (items 11 to 15 in Part 1) and knowledge items (items 4 to 6 in Part 2) have the potential to be removed to make the questionnaire more concise and able to be completed in a shorter period. This notion warrants further investigation into the content of the relevance of these items and their contribution to the overall questionnaire.

The validated DSRBQ can be used in people from a Chinese cultural background living in Australia. Further testing of the questionnaire could be performed with a different cohort as the questionnaire may be adapted for use with other Chinese or Asian diaspora groups. The validated DSRBQ will assist nurses to collect relevant data to develop a culturally based nursing assessment tool and interventions for individuals of Chinese descent who reside in a country where Chinese cultural practices are employed by a minority.

### Limitations

This study has some limitations. First, the study used a convenience sample. The findings may only represent a group of individuals of Chinese descent in Australia who are single, young, well-educated and mostly females, with no hypertension-related medical conditions. This may restrict the extent to which the results are generalisable to Australians of Chinese ancestry, especially for older people living with hypertension and required to follow a low-salt diet for secondary prevention. However, the participants were recruited through social media, resulting in a diverse sample of participants from different states and territories in Australia. Second, although a reminder was sent to all participants, only 34% of the participants completed the retest (T2) in Week 2. This might have had some impact on the test–retest reliability results, and this possibility should be considered when assessing the suitability of the measure for use in future studies.

## Conclusions

The DSRBQ has been revised and translated into English. The psychometric testing results indicate that both the English and Chinese versions have acceptable validity and reliability. The tools can be used in people from a Chinese cultural background living in Australia. With further testing, the tools may have potential for use with other Chinese or Asian diaspora groups. The findings from the tools will provide evidence about the health risks and education needs associated with dietary salt consumption in this population group. Such evidence will assist stakeholders to develop cultural appropriate community-based interventions to reduce salt intake for Chinese diaspora groups.

## Data Availability

All data generated or analyzed during this study are included in this published article.

## Abbreviations

CVI: Content validity index
COVID-19: Coronavirus
DSRBQ: Determinants of Salt-Restriction Behaviour Questionnaire
HBM: Health belief model
ICC: Intraclass correlation coefficient
I-CVI: Item-level content validity index
S-CVI: Scale-level content validity index
SD: Standard deviation
STROBE: STrengthening the Reporting of OBservational studies in Epidemiology
T1: First test
T2: Retest
WHO: World Health Organization

## References

[1] World Health Organization. Guideline: Sodium intake for adults and children. Geneva: WHO; 2012. 23658998

[2] World Health Organization. A global brief on hypertension: Silent killer, global publichealth crisis. Geneva: WHO; 2013.

[3] Mente A, O'Donnell M, Rangarajan S, Dagenais G, Lear S, McQueen M (2016). Associations of urinary sodium excretion with cardiovascular events in individuals with and without hypertension: a pooled analysis of data from four studies. Lancet.

[4] de Brito-Ashurst I, Perry L, Sanders TAB, Thomas JE, Dobbie H, Varagunam M (2013). The role of salt intake and salt sensitivity in the management of hypertension in South Asian people with chronic kidney disease: a randomised controlled trial. Heart.

[5] Bi Z, Liang X, Xu A, Wang L, Shi X, Zhao W (2014). Hypertension prevalence, awareness, treatment, and control and sodium intake in Shandong Province, China: Baseline results from Shandong-Ministry of Health Action on Salt Reduction and Hypertension (SMASH), 2011. Prev Chronic Dis.

[6] Oh VM (2016). Dietary salt intake and hypertension in Singapore. J Hypertens.

[7] Lee M, Hu D, Bunney G, Gadegbeku CA, Edmundowicz D, Houser SR (2018). Health behavior practice among understudied Chinese and Filipino Americans with cardiometabolic diseases. Prev Med Rep.

[8] Poston DL, Wong JH (2016). The Chinese diaspora: The current distribution of the overseas Chinese population. Chinese J Sociol.

[9] Australian Bureau of Statistics. ABS reveals insights into Australia’s Chinese population on Chinese New Year. Canberra: Australian Bureau of Statistics; 2018. Available from: https://www.abs.gov.au/AUSSTATS/abs@.nsf/mediareleasesbytitle/D8CAE4F74B82D446CA258235000F2BDE.

[10] Jin K, Gullick J, Neubeck L, Koo F, Ding D (2017). Acculturation is associated with higher prevalence of cardiovascular disease risk-factors among Chinese immigrants in Australia: Evidence from a large population-based cohort. Eur J Prev Cardiol.

[11] Zhang P, He FJ, Li Y, Li C, Wu J, Ma J (2020). Reducing salt intake in China with "Action on Salt China" (ASC): Protocol for campaigns and randomized controlled trials. JMIR Res Protoc.

[12] Yu YH, Farmer A, Mager D, Willows N (2014). Dietary sodium intakes and food sources of sodium in Canadian-born and Asian-born individuals of Chinese ethnicity at a Canadian university campus. J Am Coll Health.

[13] Cappuccio FP, Chen J, Donfrancesco C, Palmieri L, Ippolito R, Vanuzzo D (2015). Geographic and socioeconomic variation of sodium and potassium intake in Italy: Results from the MINISAL-GIRCSI programme. BMJ Open.

[14] Grimes CA, Khokhar D, Bolton KA, Trieu K, Potter J, Davidson C (2020). Salt-related knowledge, attitudes and behaviors (KABs) among Victorian adults following 22-months of a consumer awareness campaign. Nutrients.

[15] Mente A, O'Donnell M, Yusuf S. Sodium Intake and Health: What Should We Recommend Based on the Current Evidence? Nutrients. 2021;13(9). 10.3390/nu13093232 10.3390/nu13093232 PMC8468043 34579105

[16] Chan A, Chan SWC, Khanam M, Kinsman L (2022). Factors affecting reductions in dietary salt consumption in people of Chinese descent: an integrative review. J Adv Nurs.

[17] Chen J, Tian Y, Liao Y, Yang S, Li Z, He C (2013). Salt-restriction-spoon improved the salt intake among residents in China. PLoS One.

[18] Chen J, Liao Y, Li Z, Tian Y, Yang S, He C (2013). Determinants of salt-restriction-spoon using behavior in China: Application of the Health Belief Model. PLoS One.

[19] Chen J, Liao YX, Li ZT, Tian Y, Yang SS, Tu DH (2014). Analysis of the determinants of salt-restriction behavior among urban and rural residents in Beijing with Health Belief Model. Beijing Da Xue Xue Bao.

[20] Lim SH, He HG, Chan SWC (2017). Psychometric Properties of the Chinese Version of the Acceptance of Chronic Health Conditions (Stoma) Scale for Patients With Stoma. Cancer Nurs.

[21] Chan KS, Li HC, Chan SW, Lopez V (2012). Herth hope index: Psychometric testing of the Chinese version. J Adv Nurs.

[22] Rodrigues IB, Adachi JD, Beattie KA, MacDermid JC (2017). Development and validation of a new tool to measure the facilitators, barriers and preferences to exercise in people with osteoporosis. BMC Musculoskelet Disord.

[23] von Elm E, Altman DG, Egger M, Pocock SJ, Gøtzsche PC, Vandenbroucke JP (2007). The Strengthening the Reporting of Observational Studies in Epidemiology (STROBE) statement: Guidelines for reporting observational studies. Ann Intern Med.

[24] Jones PS, Lee JW, Phillips LR, Zhang XE, Jaceldo KB (2001). An adaptation of Brislin's translation model for cross-cultural research. Nurs Res.

[25] Girard MP, O'Shaughnessy J, Doucet C, Lardon E, Stuge B, Ruchat SM (2018). Validation of the French-Canadian pelvic girdle questionnaire. J Manipulative Physiol Ther.

[26] Gunawan J, Marzilli C, Aungsuroch Y (2021). Establishing appropriate sample size for developing and validating a questionnaire in nursing research. Belitung Nurs J.

[27] Perneger TV, Courvoisier DS, Hudelson PM, Gayet-Ageron A (2015). Sample size for pre-tests of questionnaires. Qual Life Res.

[28] Sim J, Wright CC (2005). The kappa statistic in reliability studies: Use, interpretation, and sample size requirements. Phys Ther.

[29] Ghisi GLM, Sandison N, Oh P (2016). Development, pilot testing and psychometric validation of a short version of the coronary artery disease education questionnaire: The CADE-Q SV. Patient Educ Couns.

[30] Perinetti G (2018). StaTips Part IV: Selection, interpretation and reporting of the intraclass correlation coefficient. South Eur J Orthod Dentofac Res.

[31] Vaske JJ, Beaman J, Sponarski CC (2017). Rethinking internal consistency in Cronbach's alpha. Leis Sci.

[32] Australian Bureau of Statistics. Household income and wealth, Australia. In: Statistics ABo, editor. Canberra: Australian Bureau of Statistics; 2019.

[33] Polit DF, Beck CT (2006). The content validity index: are you sure you know what's being reported? Critique and recommendations. Res Nurs Health.

[34] Taber KS (2018). The use of Cronbach’s alpha when developing and reporting research instruments in science education. Res Sci Educ.

[35] Streiner DL, Norman GR, Cairney J (2015). Health measurement scales: A practical guide to their development and use.

[36] Zhang Y, Zhang H, Li S, Li Y, Hu C, Li H (2022). Development of a short-form Chinese health literacy scale for low salt consumption (CHLSalt-22) and its validation among hypertensive patients. BMC Nutrition.

[37] King J, Mitchelhill I, Fisher M (2008). Development of a congenital adrenal hyperplasia knowledge assessment questionnaire (CAHKAQ). J Clin Nurs.

[38] Zhou C, Wang Y, Wang S, Ou J, Wu Y (2019). Translation, cultural adaptation, validation, and reliability study of the Quick-EBP-VIK instrument: Chinese version. J Eval Clin Pract.

